# Harnessing mesenchymal stromal cells for liver disease therapy: from mechanistic discoveries to clinical breakthroughs

**DOI:** 10.3389/fmed.2025.1677021

**Published:** 2026-02-04

**Authors:** Yiting Huang, Ruogu Cheng, Guaijuan Wang, Zihan Xu, Shuping Liu, Yantong Wan, Xinjiang Hou, Jieyan Wang

**Affiliations:** 1 The First Clinical Medical College, Southern Medical University, Guangzhou, China; 2 Department of Urology, People’s Hospital of Longhua, Shenzhen, China; 3 College of Medicine, Xi'an International University, Xi'an, China; 4 State Key Laboratory of Cellular Stress Biology, School of Life Science, Faculty of Medicine and Life Sciences, Xiamen University, Xiamen, China

**Keywords:** clinical applications, hepatic regeneration, HSCs, immunomodulation, MSCs

## Abstract

Mesenchymal stromal cells (MSCs) demonstrate significant potential in liver tissue regeneration and disease treatment due to their unique immunomodulatory properties, multipotent differentiation capabilities, and paracrine functions. This article provides a systematic review of MSCs’ biological characteristics and mechanistic roles in liver regeneration and summarizes recent clinical advancements and future challenges. Evidence reveals that MSCs exert therapeutic effects by secreting bioactive mediators—including hepatocyte growth factor (HGF), vascular endothelial growth factor (VEGF), and extracellular vesicles (EVs)—to inhibit hepatic stellate cell (HSCs) activation, degrade fibrotic extracellular matrix(ECM), and stimulate endogenous hepatocyte proliferation coupled with neovascularization. Their immunomodulatory functions reshape the hepatic immune microenvironment through inducing macrophage polarization toward the anti-inflammatory M2 phenotype, suppressing T-cell activation, and modulating the Th17/Treg balance. Preclinical studies confirm that MSCs effectively restore liver function and reverse fibrosis in diverse liver injury models. Preliminary clinical trials further validate their safety and efficacy, with allogeneic MSC infusion demonstrating survival benefits in end-stage liver disease patients. However, heterogeneity in cell sources, low homing efficiency, and lack of standardized preparation protocols remain major bottlenecks for clinical application. Emerging strategies integrating CRISPR-based gene editing, engineered exosome delivery platforms, and biomaterial-guided localization are imperative to refine targeting specificity and therapeutic precision. This review provides theoretical support and innovative directions for the translational application of MSCs in liver disease therapy.

## 1 Introduction

Mesenchymal stromal cells (MSCs), first characterized by Friedenstein et al. (1) through bone marrow isolation, represent a class of self-renewing adult stem cells with multilineage differentiation potential (1). MSCs demonstrate broad tissue distribution, with established isolation protocols for adult-derived (bone marrow, adipose tissue, peripheral blood) and perinatal-derived (umbilical cord, placental) sources (2). Liver disease treatment presents substantial challenges stemming from multifactorial pathogenesis and therapeutic constraints. MSCs, characterized by low immunogenicity, clinical accessibility, and multilineage differentiation potential, demonstrate prominent therapeutic advantages in hepatic regeneration.

## 2 Mechanisms of MSC-mediated hepatic repair

The therapeutic efficacy of Mesenchymal Stromal Cells (MSCs) in liver disease is not driven by a solitary pathway but rather by a synergistic network that remodels the pathological microenvironment. Instead of functioning merely as cellular replacements, MSCs act as dynamic “medicinal signaling cells,” orchestrating tissue repair through four hierarchical dimensions: immunomodulation, direct anti-fibrotic intervention, promotion of regeneration, and emerging metabolic regulation.

### 2.1 Immunomodulation: reshaping the hepatic immune microenvironment

**Figure 1:**
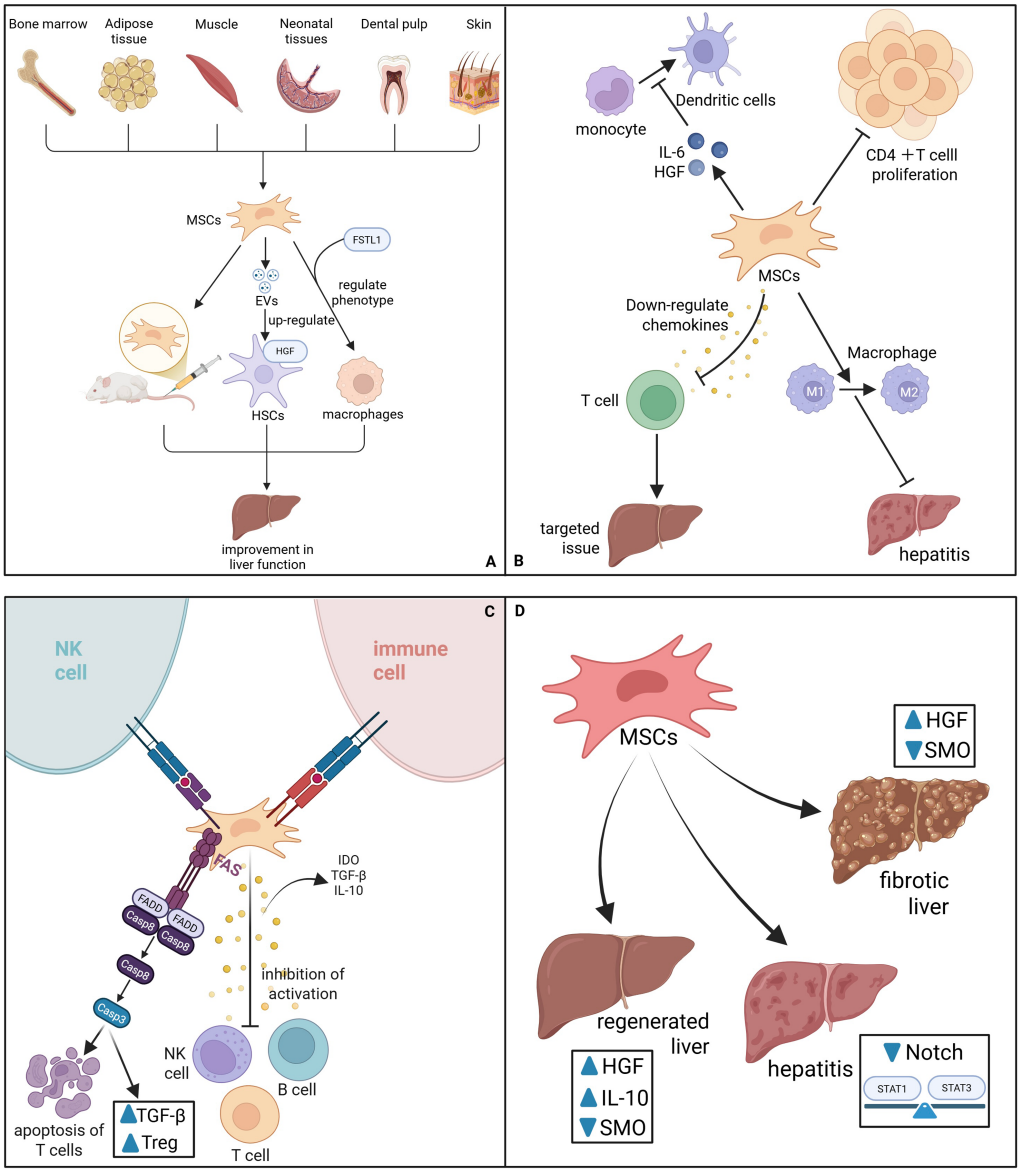
**(A)** MSCs come from a wide range of sources. When injecting MSCs into a rat liver fibrosis model, the liver function of the rats after transplantation was improved. Under the action of FSTL1, MSCs can directly rely on their strong paracrine function and immunosuppression to modulate the phenotype of macrophages, thereby mitigating the progression of liver fibrosis. MSCs-EVs can indirectly promote the upregulation of HGF expression in HSCs, thereby promoting liver regeneration. **(B)** MSCs can inhibit the proliferation of CD4 T cells and rely on TSG-6 to reduce inflammation by converting macrophages from pro-inflammatory M1 to anti-inflammatory M2. MSCs can also downregulate chemokines and inhibit the movement of T cells to target tissues. MSCs can also inhibit the differentiation of monocytes into DCs, a process that depends on their secretion of IL-6 and HGF. **(C)** MSCs express low levels of MHC class I molecules to protect them from NK cell attack. It does not express MHC class II molecules and costimulatory molecules, evading recognition by host immune cells. MHC can secrete a variety of cytokines such as IDO, TGFβ, IL-10, etc., and inhibit the activation of T cells, B cells, and NK cells. MHC induces apoptosis of T cells through Fas/FasL, upregulating Treg cells and serum TGF-β. **(D)** MSCs can increase the production of HGF and IL-10, downregulate SMO, and promote liver regeneration. MSCs may inhibit Notch pathway signaling and reverse the Stat1/Stat3 pathway imbalance, reducing pro-inflammatory cytokine levels. MSCs can also downregulate the expression of SMO, increase HGF levels, and also reverse liver fibrosis.

The inherent immunoregulatory capacity of MSCs serves as the foundation for their therapeutic value, particularly in resolving the chronic inflammation that propels liver disease progression (Figure 1A). Central to this mechanism is the modulation of macrophage plasticity; MSCs effectively shift the hepatic macrophage population from a pro-inflammatory M1 phenotype toward an anti-inflammatory and restorative M2 phenotype. This repolarization is mediated through the secretion of soluble factors such as Tumor Necrosis Factor-Inducible Gene 6 Protein (TSG-6) and Interleukin-10 (IL-10), which collectively mitigate hepatic inflammation (3–5). Under inflammatory conditions primed by IFN-*γ* and TNF, MSCs further upregulate PD-L1 and IL-10 to reinforce this immunosuppressive niche and promote the generation of regulatory T cells (Tregs) (6–9). Beyond macrophage regulation, MSCs exert potent suppressive effects on adaptive immune responses. They inhibit the activation and proliferation of CD4 + T cells by secreting indoleamine 2,3-dioxygenase (IDO) to degrade essential tryptophan (3, 4), while simultaneously downregulating chemokines such as CXCL9, CXCL10, and CXCL11 to block the trafficking of pathogenic T cells to injury sites (10). In parallel, MSC-derived extracellular vesicles (MSC-EVs) have been identified to interrupt B-cell activation cascades by inhibiting MAPK and NF-κB signaling pathways, thereby dampening the production of inflammatory mediators (11). To establish long-term tolerance, MSCs induce T-cell apoptosis via the Fas/FasL pathway and inhibit the differentiation of monocytes into dendritic cells (DCs) in an IL-6/HGF-dependent manner (12–15), creating a positive feedback loop that amplifies IL-10 production and restores immune homeostasis (16).

### 2.2 Anti-fibrotic activity: direct targeting of HSCs

As the central driver of fibrogenesis, the activated Hepatic Stellate Cell (HSC) represents a primary therapeutic target for MSCs. MSCs intervene directly in the fibrotic cascade through multiple molecular avenues to halt or reverse ECM deposition. Mechanistically, MSCs and their derived EVs (such as MEVM) directly suppress the expression of key fibrotic markers, including *α*-smooth muscle actin (α-SMA), Col1α1, and vimentin, effectively reverting activated HSCs to a more quiescent state (17, 18). This deactivation is further reinforced by the secretion of specific paracrine factors like Follistatin-like 1 (FSTL1) and HGF, which inhibit HSC proliferation and activation (19, 20). Furthermore, MSCs exert their anti-fibrotic effects by intercepting critical pro-fibrotic signaling nodes. Studies demonstrate that MSCs can downregulate the Notch signaling pathway and restore the homeostatic balance of STAT1/STAT3 signaling, thereby attenuating the inflammatory drive behind fibrosis (21). Additionally, both MSCs and their exosomes have been shown to downregulate Smoothened (SMO) expression, consequently inhibiting the Hedgehog/SMO signaling pathway. Complementing these cellular regulatory mechanisms, MSCs actively remodel the fibrotic matrix by secreting matrix metalloproteinases (MMP-1, MMP-9) while downregulating tissue inhibitors of metalloproteinases (TIMPs), thus promoting the degradation of established collagen deposits (22, 23) (Figure 1B).

### 2.3 Promotion of angiogenesis and hepatocyte regeneration

To facilitate functional recovery, MSCs provide critical trophic support that stimulates endogenous repair processes. They release a plethora of bioactive mediators, including HGF, VEGF, and EVs, which act synergistically to stimulate hepatocyte proliferation and promote neovascularization (24–27). This paracrine function is crucial in acute liver failure (ALF), where MSCs rapidly suppress inflammation while providing the necessary growth factors to arrest liver damage (28). Recent bioengineering strategies have further amplified these effects; for instance, MSCs engineered to overexpress VEGF have demonstrated superior therapeutic efficacy in protecting against acute injury (29). Moreover, epigenetic regulation via MSC-EVs has emerged as a key regenerative mechanism. Specifically, the long non-coding RNA lncEEF1G enriched in MSC-EVs has been found to act as a competitive endogenous RNA, regulating the miR-181a-5p/HGF axis to enhance HGF expression in HSCs, thereby indirectly fueling hepatocyte regeneration (19) (Figure 1C).

### 2.4 Emerging mechanisms: ferroptosis modulation and mitochondrial transfer

Recent advancements have uncovered sophisticated metabolic and organelle-level interactions between MSCs and hepatic cells, offering precise new targets for therapy. A novel cytoprotective mechanism involves the direct transfer of functional mitochondria from MSCs to injured hepatocytes via tunneling nanotubes (TNTs). This physical transfer restores Glutathione Peroxidase 4 (GPX4) activity and mitochondrial homeostasis, effectively rescuing hepatocytes from lipid peroxidation and bioenergetic failure (30). Concurrently, MSCs exhibit a dual regulatory role in ferroptosis—an iron-dependent form of cell death. While protecting hepatocytes, EVs derived from umbilical cord MSCs (UC-MSC-EVs) have been shown to selectively induce ferroptosis in activated HSCs via the exosomal BECN1 pathway. This process specifically reduces system xc^−^/GPX4 activity in fibrotic cells without compromising healthy hepatocytes (31). This cell-selective modulation represents a cutting-edge therapeutic strategy that balances hepatocyte preservation with the targeted elimination of fibrotic scar tissue (Figure 1D). Under the anti-fibrotic framework, MSCs can also further silence HSCS through the ‘ferroptosis - mitochondrial axis’. Specifically, MSCs transfer healthy mitochondria to damaged hepatocytes via tunnel nanotubes (TNT), restoring their GPX4 activity and inhibiting lipid peroxidation, thereby blocking the activation of HSC driven by ferroptosis (Figure 1D). This mechanism is a supplement to the aforementioned TGF-*β*/STAT pathway rather than an independent event. Moreover, UC-MSC-EVs specifically induce ferroptosis in the human HSCs line LX2 (without affecting hepatocytes) via exosomal BECN1, which reduces system xc^−^/GPX4 activity—an effect weakened by BECN1 knockdown (31). In liver fibrosis, they target activated HSCs via this pathway to reverse scarring, and their cell-selective modulation avoids systemic iron disturbance and off-target hepatocyte damage, showing clinical promise for targeted liver repair.

## 3 Preclinical evidence and mechanistic insights of MSCs-based hepatic therapy

### 3.1 Pathological challenges in hepatic injury and regeneration

Hepatic injuries are clinically categorized into metabolic, viral, immune-mediated, and genetic liver disorders. Metabolic dysfunction-associated steatotic liver disease (MASLD), driven by insulin resistance, triggers lipid accumulation and oxidative stress, which can progress to metabolic dysfunction-associated steatohepatitis (MASH) and fibrosis. Without intervention, MASH may advance to fibrosis, ultimately culminating in cirrhosis or hepatocellular carcinoma (HCC) (32–34). Viral hepatitis (HBV/HCV) and drug-induced liver injury constitute primary etiologies of acute liver failure (ALF), pathologically characterized by rapid hepatocyte necrosis and hepatic decompensation, with 28-day mortality exceeding 15% (35, 36). Autoimmune hepatitis involves chronic inflammation and fibrosis from aberrant immune attacks, whereas inherited disorders like Wilson’s disease directly damage hepatocytes through metabolic defects (37, 38). Additionally, biliary atresia caused by bile duct developmental abnormalities leads to cholestasis, resulting in progressive neonatal liver injury and infantile cirrhosis (39, 40).

**Figure 2:**
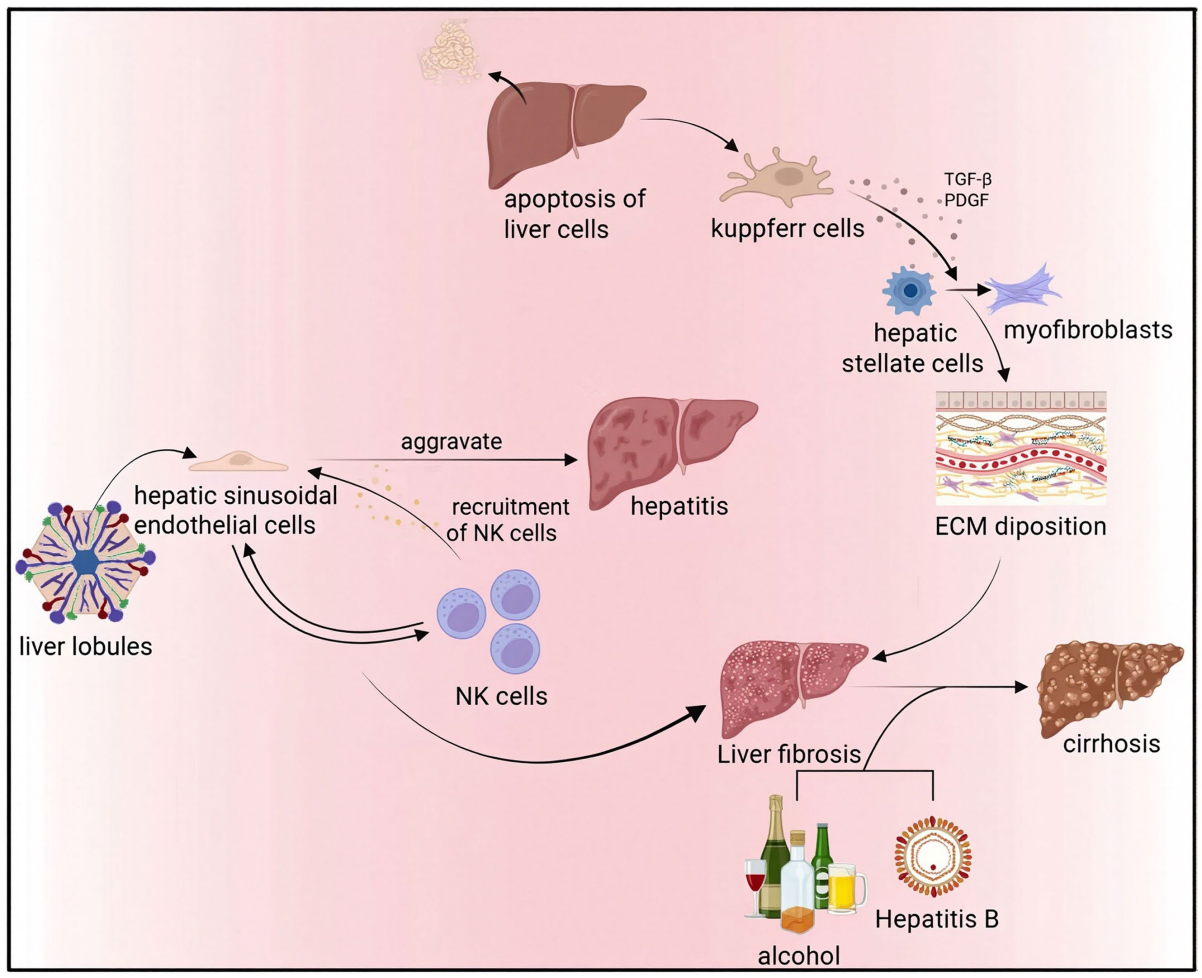
Hepatocyte apoptosis triggers Kupffer cells (KCs) to release TGF-β and PDGF, activating HSCs to differentiate into myofibroblasts, causing excessive extracellular matrix (ECM) deposition. Liver sinusoidal endothelial cells exacerbate inflammatory responses by recruiting natural killer (NK) cells, while interactions between immune cells and hepatic parenchymal cells further drive fibrosis progression. Persistent insults like alcohol abuse or chronic viral infection ultimately result in cirrhosis with architectural disruption and irreversible loss of regenerative potential.

The hepatic regenerative capacity is profoundly compromised by chronic injury. Fibrogenesis constitutes the central pathological cascade. This process initiates when hepatocyte apoptosis triggers Kupffer cells to release TGF-*β* and PDGF. These cytokines then activate HSCs, prompting their differentiation into myofibroblasts. The primary consequence of this activation is the excessive deposition of ECM (41). Liver sinusoidal endothelial cells exacerbate inflammatory responses by recruiting natural killer (NK) cells, while interactions between immune cells and hepatic parenchymal cells further drive fibrosis progression (42). Although early-stage fibrosis remains reversible, persistent insults (e.g., alcohol abuse or chronic viral infection) ultimately result in cirrhosis with architectural disruption and irreversible loss of regenerative potential (43, 44) (Figure 2).

**Figure 3:**
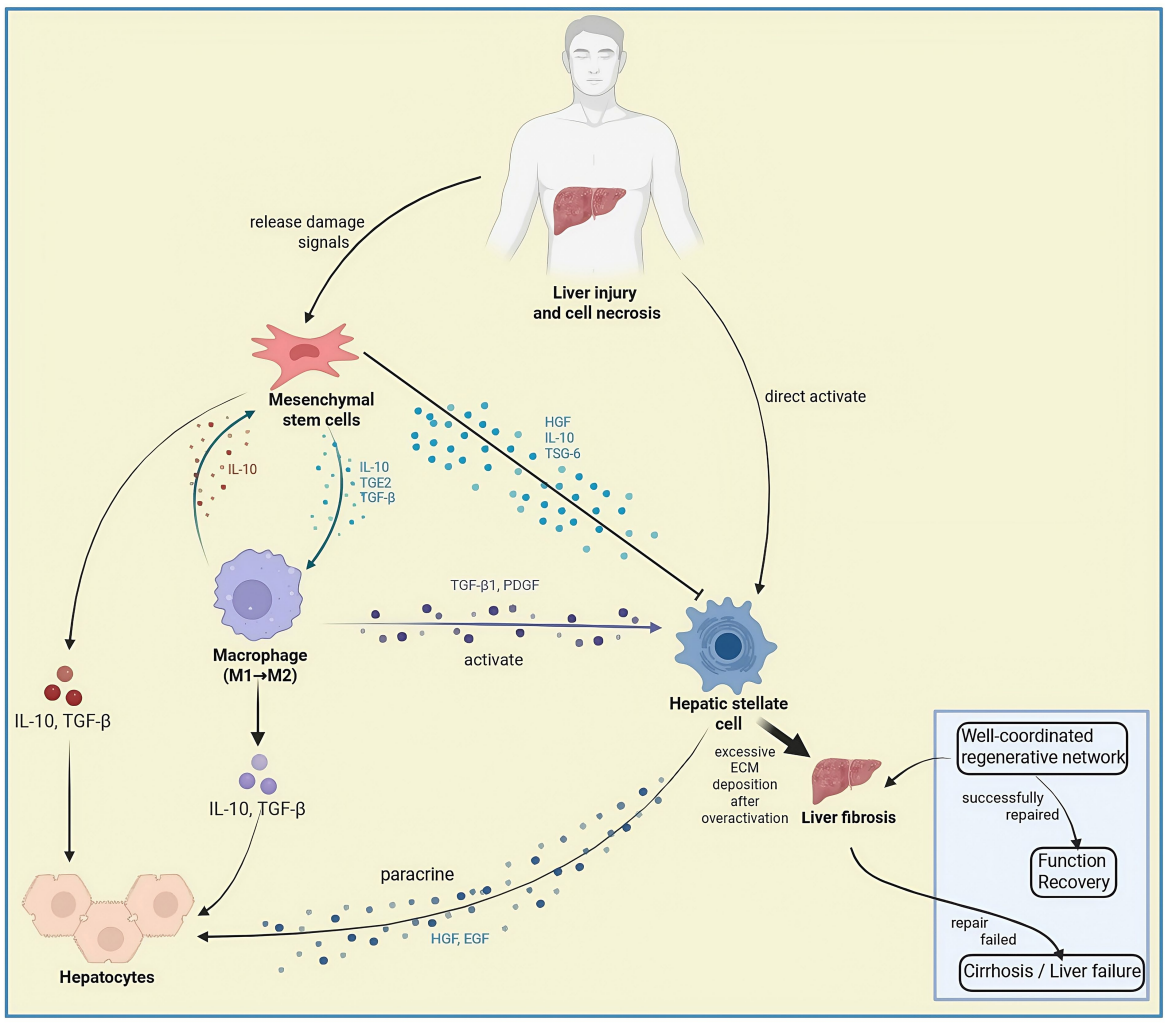
This diagram illustrates the cellular crosstalk during liver regeneration following injury. Mesenchymal stem cells (MSCs) modulate macrophage polarization toward M2 phenotype (via IL-10, TGF-β) and suppress hepatic stellate cell activation (via HGF, IL-10, TSG-6). Immune cells activate stellate cells (via TGF-β1, PDGF) while initiating hepatocyte proliferation (via TNF-*α*, IL-6). Hepatic stellate cells (HSCs) contribute to regeneration through paracrine signaling but driving fibrosis via excessive ECM deposition when overactivated. The balance between the pro-regenerative network and pro-fibrotic signals determines the pathological outcome.

Significant etiological heterogeneity exists across liver diseases. Although MASLD and viral hepatitis differ etiologically, both converge on HSCs activation to drive end-stage pathology (45). Therapeutic options remain inadequate for advanced-stage conditions including decompensated cirrhosis and acute liver failure. Liver transplantation, despite being the sole curative modality, faces critical limitations encompassing donor scarcity, immunological rejection risks, and substantial economic burdens (46). Current therapeutic strategies inadequately address multicellular interaction networks, particularly the oxidative stress-inflammation vicious cycle in MASLD and the regenerative microenvironment disruption in ALF (47, 48). The rapid progression of ACLF renders conventional supportive therapies ineffective in improving prognosis (49). Furthermore, therapeutic gaps persist in managing cirrhosis-related complications such as portal hypertension, where mechanism-specific interventions are urgently needed. These unresolved challenges underscore the imperative for precise strategies for fibrosis reversal and hepatocyte regeneration (Figure 3).

### 3.2 Preclinical liver disease models for MSC research

Animal models have become indispensable in preclinical investigations of MSCs-based therapies for hepatic injury, particularly in elucidating cellular repair mechanisms. Investigators have developed multimodal liver disease models employing chemical induction, surgical manipulation, and genetic engineering strategies that recapitulate human hepatic pathophysiology. CCl4- and DEN-induced models reliably recapitulate hepatic fibrogenesis (50), whereas Con-A models are preferentially utilized to delineate immune homeostasis disruption and inflammatory cytokine network (51). The BDL model is commonly used to study cholestasis-related fibrosis, exhibiting pathomorphological characteristics analogous to clinical biliary obstruction (52). Genetically modified animal models provide precise research platforms for liver injury caused by viral hepatitis or specific genetic defects (53, 54).

Porcine ALF models, with their human-like hepatic cytoarchitecture and metabolic profiles, serve as critical translational platforms for MSCs biodistribution and therapeutic efficacy assessments (55, 56). Portal venous MSCs administration in porcine ALF models demonstrates superior engraftment efficiency and hepatoprotective effects compared to peripheral delivery routes, as evidenced by enhanced functional recovery and survival outcomes (57–59).

To guide preclinical studies of MSCs-based therapy for hepatic injury, targeted model selection is critical: small animal models should align with specific research goals (e.g., anti-fibrotic efficacy, immunomodulatory mechanism), while large animal models are indispensable for verifying MSCs biodistribution, optimizing delivery routes, and narrowing the preclinical-clinical translational gap.

Although existing clinical trials have confirmed the safety and preliminary efficacy of MSCs in the treatment of liver diseases, the results show significant heterogeneity, which suggests that more detailed clinical evaluations and stratified analyses are needed. Firstly, the differences among various patient groups are of crucial importance: disease stage (such as compensated and decompensated liver cirrhosis), etiology (viral, alcoholic, metabolism-related), comorbidities (such as diabetes, immune function status), and individual immune background may all affect the response effect of MSCs. For instance, in patients with decompensated liver cirrhosis, the function of autologous MSCs may be impaired. At this time, allogeneic MSCs may have more advantages, and patients with early liver fibrosis may be more sensitive to the immunomodulatory and anti-fibrotic effects of MSCs.

### 3.3 Therapeutic potential of MSCs in liver regeneration

The unique biological properties of MSCs encompassing anti-inflammatory, anti-fibrotic, and immunomodulatory functions establish MSCs as ideal candidates for reversing hepatic injury and facilitating tissue repair. The anti-fibrotic effects involve MSCs-mediated collagen degradation via MMP-1/MMP-9 secretion and direct suppression of HSCs activation (22, 23). Clinical trial (NCT01741090) documented significant reduction in Laennec fibrosis scores following autologous BM-MSCs administration in cirrhotic patients. Paracrine mechanisms through HGF and VEGF secretion stimulate endogenous hepatocyte proliferation and angiogenesis, correlating with improved 24-week survival rates (73.2%) in HBV-associated ACLF patients (60). Immunomodulatory functions are achieved through TSG-6/IL-10-mediated suppression of neutrophil infiltration and NF-κB signaling, coupled with macrophage polarization toward anti-inflammatory M2 phenotype to remodel hepatic immune microenvironment (61–63). Current challenges for MSCs therapy include suboptimal homing efficiency and cellular heterogeneity. To address these issues, researchers are optimizing delivery routes, such as portal vein infusion, and employing genetic modifications, like Smad7 overexpression. These strategies aim to enhance the targeting precision of MSCs (64). Collectively, MSCs provide an innovative therapeutic paradigm for end-stage liver disease through synergistic integration of anti-fibrotic, regenerative, and immunoregulatory mechanisms.

In compensated cirrhosis, autologous transplantation can avoid rejection reactions and has lower immunological risks, making it a preferable option; whereas in decompensated cirrhosis, the patient’s own stem cell function is impaired, making allogeneic stem cell transplantation more advantageous. As shown in the table above, hepatitis and cirrhosis are mostly treated with autologous transplantation, while liver failure is primarily managed with allogeneic stem cell transplantation (Table 1).

**Table 1:** Clinical trials of MSCs therapy for liver diseases.

Disease type	Cell source	Administration route	Ample size	Primary endpoint indicator	Results	References
Hepatitis B	Autologous bone MSCs	Hepatic artery injection	Stem cell group (*n* = 53)/Control group (*n* = 105)	Albumin, total bilirubin, prothrombin time levels, and MELD score	The patients in the transplantation group showed significantly higher levels of albumin, total bilirubin, prothrombin time, and MELD score 2 to 3 weeks after transplantation compared to the control group. During the 192-week follow-up period, there was no significant difference in the incidence and mortality of hepatocellular carcinoma between the two groups. Additionally, in the transplantation group, there was no significant difference in the incidence and mortality of HCC between patients with and without liver cirrhosis.	Peng et al. (125)
Hepatitis C	Autologous BM-MSCs	Intravenous injection	Stem cell group (*n* = 15)/Control group (*n* = 10)	Prothrombin concentration and serum albumin level, high bilirubin, and MELD score	The liver function tests in the MSCs group showed partial improvement, with an increase in prothrombin concentration and serum albumin levels, and a decrease in bilirubin levels and MELD score.	El-Ansary et al. (126)
Hepatitis b cirrhosis	Autologous BM-MSCs	Hepatic artery injection	Stem cell group (*n* = 20)/Control group (*n* = 19 cases)	End-stage liver disease score, liver function	Although both groups of patients showed significant improvement after receiving entecavir treatment, BM-MSCs transplantation further enhanced the liver function of the patients.	Xu et al. (127)
Alcoholic cirrhosis	Autologous BM-MSCs	Hepatic artery injection	Control group (*n* = 24)/Single infusion group (*n* = 24)/Double infusion group (*n* = 24)	The primary endpoint was the improvement in fibrosis score, while the secondary endpoints were liver function, Child-Pugh score, and MELD score.	Compared with the control group, the fibrosis score and Child-Pugh score of MSCs showed significant improvement at the 24th week, but there was no statistically significant difference between the single infusion group and the double infusion group.	Suk et al. (71)
Liver cirrhosis of various etiologies	Allogeneic BM-MSCs	peripheral vein injection	Stem cell group (*n* = 4)/Control group (*n* = 5)	Child-Pugh, Model for End-Stage Liver Disease, and Acute-on-Chronic Liver Failure	The 90-day survival rate of the placebo group was 20% (1/5), while that of the BM-MSCs group was 25% (1/4). Patients who completed the entire MSCs protocol showed significant improvements in CP (C-14 to B-9), MELD (32 to 22), and ACLF (3 to 0 grades).	Schacher et al. (75)
Acute liver failure related to the hepatitis B virus	Allogeneic BM-MSCs	Peripheral vein injection	Stem cell group (*n* = 56)/Control group (*n* = 54)	Serum total bilirubin and MELD score	Peripheral infusion of allogeneic BM-MSCs is a safe and convenient treatment option for patients with HBV-related ACLF. By improving liver function and reducing the incidence of severe infections, it significantly increases the 24-week survival rate.	Lin et al. (74)
Acute liver failure related to the hepatitis B virus	Allogeneic hUC-MSCs	Hepatic artery injection	Stem cell group (*n* = 11)/Control group (*n* = 34)	Albumin, alanine aminotransferase, aspartate aminotransferase, total bilirubin, direct bilirubin, PT, INR, and end-stage liver disease model score	For patients with hepatitis B-related acute liver failure who receive treatment with pegylated interferon combined with entecavir, hUC-MSCs transplantation is safe and effective. This therapy can further enhance liver function and improve the survival rate of patients.	Lin et al. (74)

hUC-MSCs, human umbilical cord-derived mesenchymal stem cells; MELD, Model for End-Stage Liver Disease; PT, prothrombin time; INR, international normalized ratio.

### 3.4 Source-dependent and strategy-dependent preclinical outcomes

Further studies have found that the reparative effects of MSCs are closely related to cell sources and treatment strategies. BM-MSCs exhibit superior antifibrotic potential in murine hepatic fibrosis models, mediated through suppression of TGF-*β* signaling and HSCs deactivation (65). Allogeneic MSCs demonstrate reduced therapeutic potency compared to autologous counterparts in acute injury settings, despite being easily accessible (66). MSC-EVs have emerged as promising therapeutic vectors, with meta-analyses confirming their monotherapeutic capacity to attenuate fibrogenesis and improve hepatic function. Synergistic effects are observed when combined with standard antifibrotics, particularly in intraperitoneally administered AD-MSC-EVs regimens (67, 68). These advancements substantiate MSCs-based regenerative mechanisms while providing the basis for source selection and dosing optimization, thereby accelerating translational development of cell-based hepatic therapies.

## 4 Clinical translation and challenges of MSCs-based hepatic therapy

### 4.1 Overview of clinical trials

MSCs-based clinical trials now encompass major hepatic pathologies, including cirrhosis, viral hepatitis, alcoholic liver disease, liver failure, and liver transplantation complications. While BM-MSCs, UC-MSCs, and AD-MSCs are all utilized, UC-MSCs have emerged as the predominant source owing to their inherent low immunogenicity, easy accessibility, and standardized preparation. Administration routes are mainly intravenous or targeted hepatic artery/portal vein infusion, with dosage ranging from 5 × 10⁵ to 5 × 10^7^ cells/kg. Trial designs are mostly single-arm or RCTs, with primary endpoints including liver function biomarkers (ALT, AST, Alb), Child-Pugh score, MELD score, and fibrosis markers (HA, LN) (69). In the NCT03838250 phase II trial for alcoholic cirrhosis, autologous BM-MSCs recipients achieved 2-3-point reductions in Child-Pugh scores and 15–20% serum albumin elevation, with long-term follow-up (75 months) confirming survival improvement (70, 71). MSCs combined with antiviral therapy in viral hepatitis significantly reduces inflammatory cytokines (IL-6, TNF-*α*), though without affecting viral load (72). The NCT01224327 trial established that allogeneic UC-MSCs hepatic artery infusion significantly enhances coagulation parameters and reduces MELD scores in end-stage disease, showing comparable efficacy with autologous MSCs therapy (73). The NCT01322906 trial reported 73.2% 24-week survival with allogeneic BM-MSCs, significantly better than standard care (55.6%), but requires a plasma exchange combination for enhanced efficacy (74). Safety profiles across trials show over 90% of trials reported only transient fever (5–10%) or injection-site discomfort, with no tumorigenicity evidence (74, 75). However, significant efficacy heterogeneity persists, potentially attributable to cell source variability, patient status (e.g., cirrhosis stage), and lack of consensus on dose standardization. Future requires large-scale multicenter RCTs to optimize combination therapies (e.g., MSC-EVs, gene editing) and establish long-term follow-up mechanisms (76).

### 4.2 Current status and challenges of MSCs therapy

The clinical application of MSCs in liver disease treatment shows potential but faces challenges including source heterogeneity, treatment standardization, and safety. MSCs biological characteristics differ significantly by tissue source (BM, adipose, UC): BM-MSCs tend to differentiate into osteocytes due to epigenetic memory, while UC-MSCs prefer adipogenic differentiation (77), limiting hepatic differentiation efficiency (78). To address source limitations, iPSC technology is used for unlimited MSCs expansion, but standardized preparation remains a key obstacle (79).

Traditional isolation methods (e.g., differential adhesion) easily contaminate other bone marrow cells (80, 81), while FACS/MACS can enrich specific subsets (e.g., CD105+/CD73+) but lack functional validation (82). While ISCT minimal criteria define MSCs identity, divergent culture conditions (e.g., serum sources, oxygen tension) induce profound functional heterogeneity despite phenotypic conformity (83).

Optimization of dosage and infusion routes requires consideration of disease stage and safety. Portal vein or hepatic artery injection enhances liver targeting but its invasiveness limits application (84, 85). Genetic engineering (e.g., CXCL9 overexpression) is used to enhance homing ability (86), but industrial production is limited by cell stability and regulatory compliance (87, 88).

Regarding safety, MSCs therapy mainly causes transient fever and injection reactions, with no severe infections or deaths reported (89, 90). However, the theoretical risks of tumorigenicity in genetically modified MSCs products still require monitoring (91). Significant interpatient response heterogeneity correlates strongly with hepatitis type, fibrosis stage, and immune status, requiring pathology-based stratified therapy (92, 93). Additionally, high costs, from GMP production and personalized protocols, limit accessibility in low/middle-income countries (93), requiring automated expansion, standardized cell bank sharing, and policy support (e.g., insurance coverage) to promote equitable treatment (94).

Furthermore, the broad clinical translation of MSCs-based therapies hinges on establishing rigorous manufacturing quality control and ethical oversight. The production process must ensure sterility, purity, viability, and genetic stability of the cells, particularly when using genetically modified or iPSC-MSCs. Although no severe immune rejection or tumorigenic events have been reported in existing clinical trials, long-term safety must be further validated through large-scale, multicenter studies with extended follow-up. Ethically, it is essential to ensure fully informed consent from patients, especially when using genetically engineered or allogeneic MSCs products. Additionally, the high cost of therapy may limit accessibility in resource-limited settings, necessitating policy support and technical standardization to promote equitable treatment access.

### 4.3 The differences between different sources of MSCS or MSC-related treatment methods

The therapeutic landscape of mesenchymal stem cells (MSCs) has evolved into a diversified matrix comprising native MSCs, MSC-derived extracellular vesicles (MSC-EVs), genetically engineered MSCs, and induced pluripotent stem cell–derived MSCs (iPSC-MSCs). As the cornerstone of this field, native MSCs function as living therapies capable of dynamic immunosensing and adaptive paracrine responses. They currently possess the most advanced clinical track record, supported by established GMP manufacturing workflows; however, their application is constrained by inherent challenges such as pulmonary entrapment, ectopic homing, donor-to-donor variability, and uncertain long-term persistence (95, 96). Conversely, MSC-EVs have emerged as a promising cell-free modality that recapitulates the paracrine benefits of MSCs through the delivery of bioactive cargos—including miRNAs, proteins, and lipids—while mitigating risks associated with live cell engraftment. Although EVs offer superior storage stability and a reduced tumorigenic profile, they currently occupy a regulatory “gray zone” and face significant hurdles regarding scalable production, batch-to-batch consistency, and cargo standardization (97, 98).

To address the functional and supply limitations of primary cells, genetically engineered MSCs and iPSC-MSCs represent advanced platforms for precision medicine and standardization. Genetically modified MSCs are designed to overexpress or silence specific targets (e.g., HGF, IL-10, CXCR4), thereby significantly enhancing homing efficiency and therapeutic potency. Nevertheless, these modifications introduce complex safety concerns, including insertional mutagenesis and transgene instability, subjecting them to stringent regulatory oversight comparable to gene therapy products (99, 100). Simultaneously, iPSC-MSCs provide a theoretically unlimited and standardized cell source, effectively overcoming the donor scarcity and age-related functional decline observed in primary MSCs. While iPSC-MSCs exhibit rejuvenated phenotypes and high proliferative capacity, residual risks regarding genomic instability, epigenetic abnormalities, and potential tumorigenicity necessitate rigorous quality control, keeping them at an earlier translational stage compared to their primary counterparts (79, 101).

Collectively, these four platforms should be viewed as complementary strategies rather than competing modalities. Native MSCs remain the most viable option for near-term clinical application; MSC-EVs provide a safer, acellular alternative for specific indications; genetically engineered MSCs enable high-precision interventions; and iPSC-MSCs offer a scalable solution for long-term manufacturing needs. Optimizing clinical translation requires a nuanced, indication-specific approach that carefully balances therapeutic efficacy with safety profiles, manufacturing feasibility, and regulatory compliance.

### 4.4 Emerging strategies for promoting clinical translation

#### 4.4.1 MSC-derived extracellular vesicles: translational opportunities

**Figure 4:**
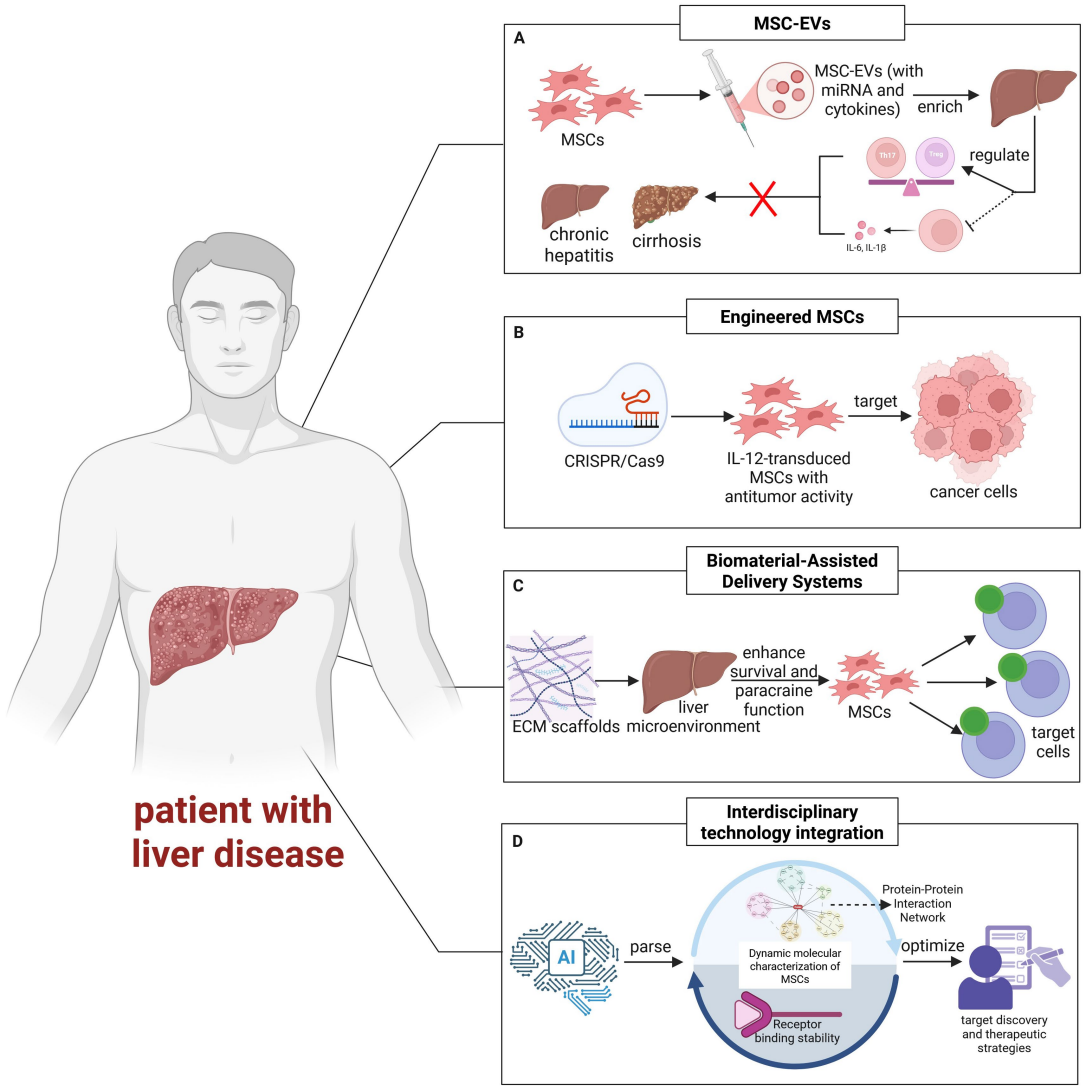
**(A)** MSC-EVs carry miRNA, cytokines, and other bioactive molecules, regulate liver immune homeostasis, inhibit the release of pro-inflammatory factors (such as IL-6 and IL-1β), and regulate Th17/Treg cell balance, thereby alleviating inflammation and fibrosis in chronic liver disease. **(B)** Gene modification of MSCs by CRISPR/Cas9 technology significantly enhanced their functional properties. IL-12 engineered MSCs showed antitumor activity in liver cancer models. **(C)** Biomaterials such as acellular ECM scaffolds can improve the survival rate of MSCs and promote paracrine function by mimicking the liver microenvironment. **(D)** Artificial intelligence (AI) accelerates target discovery and therapeutic strategy optimization by elucidating the dynamic molecular characteristics of MSCs (e.g., protein interaction networks and receptor binding stability).

MSC-EVs act as key mediators of MSCs paracrine effects. They regulate liver immune homeostasis by carrying bioactive molecules, including miRNAs and cytokines. This process suppresses pro-inflammatory factors such as IL-6 and IL-1*β*, while balancing Th17/Treg cell ratios, ultimately alleviating chronic liver inflammation and fibrosis (102, 103). Studies reveal that intravenously administered MSC-EVs predominantly accumulate in the liver, whose small size (<200 nm) facilitates tissue barrier penetration (104, 105). This characteristic demonstrates therapeutic potential in cirrhosis and steatohepatitis models (Figure 4A). Early-phase trials (ChiCTR2300075676) have preliminarily confirmed safety. Nevertheless, large-scale efficacy validation remains to be achieved (98).

In contrast to naturally secreted MSC-EVs, which inherently exhibit favorable biodistribution and low immunogenicity, engineered EVs are purposefully modified to augment their therapeutic precision and efficacy (98). Such modifications include surface functionalization with targeting ligands (e.g., RGD or GE11 peptides) to improve tissue-specific delivery, or loading with nucleic acids such as siRNA or lncRNA to manipulate gene expression in recipient cells (19). These strategic enhancements underscore the potential of engineered EVs as next-generation acellular therapeutics with improved targeting and regenerative capabilities.

#### 4.4.2 Engineered MSCs and biomaterial-assisted delivery systems

CRISPR/Cas9-based genome editing enables precise modulation of MSCs functional characteristics (100, 106). For example, IL-12-transduced MSCs demonstrate antitumor activity in HCC models (107) (Figure 4B). CRISPR/Cas9-mediated knockout of Smad7 (a negative regulator of the TGF-β pathway) in MSCs boosts TGF-β secretion, thereby enhancing their TGF-β-dependent remodeling of the hepatic immune microenvironment (108). iPSC-MSCs, owing to their replicative stability and amenability to pre-editing strategies, represent potential platforms for scaled applications (109).

Additionally, biomaterials enhance MSCs survival and paracrine function by mimicking liver microenvironment. MSCs spheroids secrete extracellular vesicles (SpEVs) enriched with bioactive proteins, which exhibit higher endocytosis efficiency by recipient hepatic cells and enhanced pro-angiogenic/antioxidative effects compared to EVs from dissociated MSCs. When combined with biomaterials like decellularized ECM hydrogels—carriers that prolong SpEV retention in the hepatic microenvironment and mimic physiological niches—this system synergistically boosts the therapeutic efficacy of engineered MSCs, particularly in ameliorating ALF via reinforced paracrine and immunomodulatory functions. However, it requires balancing stiffness and immunogenicity to avoid foreign body reactions (19, 110) (Figure 4C). Although genetic modification and material engineering enable personalized therapy, their long-term safety (e.g., insertional mutagenesis risks) still requires systematic evaluation (111–113). Two major bottlenecks hinder clinical implementation: First, standardized EV isolation and characterization protocols require development, including uniform culture volumes and isolation methods. Second, CRISPR-Cas9-based engineering for targeted gene delivery also needs optimization to enhance treatment precision.

#### 4.4.3 Interdisciplinary integration for clinical innovation

AI accelerates target discovery and therapeutic optimization by analyzing MSCs dynamic molecular features, such as protein interaction networks and receptor binding stability (114) (Figure 4D). For instance, AI addresses the critical bottleneck of MSCs heterogeneity by integrating culture-derived multi-omics data to automatically adjust parameters (e.g., optimizing hypoxic preconditioning to 12–16 h for enhanced HGF secretion) and screen high-efficacy batches, improving GMP production consistency. Nanotechnology improves liver targeting via engineered EVs (e.g., surface-modified CXCR4/TRAIL), while machine perfusion (MP) combined with EV acellular therapy directly repairs transplant organs, reducing ischemia–reperfusion injury (115–118). However, clinical translation of these technologies relies on GMP-standardized production and multidisciplinary validation mechanisms (119–121). At the same time, treatment can also be combined with other therapies such as artificial liver and engineered exosomes based on the patient’s disease type and severity, in order to establish a personalized treatment plan and achieve better and more innovative therapeutic outcomes. Future needs focus on improving clinical adaptability of AI prediction models and the efficacy-safety evaluation of cross-technology integration (122).

## 5 Conclusion

Current evidence indicates that the most core and reproducible mechanism of mesenchymal stem cell (MSC) treatment for liver diseases is: 1. Inhibit the activation of hepatic stellate cells and induce their apoptosis through paracrine factors such as HGF, VEGF, and IL-10. 2. Polarized the macrophage population from pro-inflammatory M1 to anti-inflammatory M2. 3. Up-regulated MMP-1/2/13 and down-regulated TIMP-1 to degrade the deposited collagen. Thus, it consistently reduced the indicators of *α*-SMA, TGF-β1, collagen III and serum liver fibrosis in different animal models and multiple phase I/II trials, and improved the synthetic functional parameters such as ALB, ALT and AST (123).

In contrast, the strategies of MSCS “directly differentiating into functional hepatocytes” to supplement liver parenchyma and “combining with drugs or genetic modifications to enhance homing rates” currently only show additional benefits in small samples or specific models, and there is still a lack of multi-center randomized controlled evidence. Moreover, the efficacy differences of MSCS from different tissues, the optimal infusion timing and routes, as well as the long-term tumorigenicity/immune risks remain unclear. Therefore, their clinical value still depends on specific experimental conditions and is regarded as weak evidence support (95).

The triple mechanism of MSCs-mediated immunomodulation, multilineage differentiation capacity, and paracrine signaling orchestrates hepatic repair through inhibiting HSCs activation, promoting hepatocyte regeneration, and niche remodeling, establishing their therapeutic superiority in fibrotic resolution. Preclinical studies confirm significant therapeutic efficacy of MSCs for liver diseases, and preliminary trials indicate safety and effectiveness, though large-scale validation is still required. Notably, compared to conventional MSCs therapy, MSC-EVs have been confirmed to offer several advantages, primarily focusing on lower immunogenicity, convenience, quality control, and ease of preparation (124). Currently, there are relatively few clinical trials studying MSC-EVs therapy for liver diseases, and comparative studies using the same model, same dose, and same treatment course are still scarce. However, critical knowledge gaps hinder their translation to routine clinical practice: the mechanism behind source-specific MSCs efficacy across different liver injury subtypes remains unclear; the long-term crosstalk between MSCs and hepatic non-parenchymal cells, as well as potential adaptive resistance in chronic injury, is understudied; and key parameters of MSC-EVs—including molecular targets, pharmacokinetics, and human dose–response relationships—remain undefined. To unlock MSCs’ full potential, future research must focus on three core directions: First, optimize cell sourcing—advancing iPSC-MSCs to resolve donor scarcity and exploring liver-resident MSCs for better hepatic compatibility. Second, improve targeting specificity—via CRISPR-mediated overexpression of liver-homing receptors, MSC-EVs surface modification, or combination with biomaterial scaffolds for local retention. Third, evaluate long-term safety—through large-scale, multi-center trials with extended follow-up (≥5 years) to assess risks like tumorigenicity of modified MSCs or chronic immune effects. Only by advancing these efforts can MSCs-based therapies become safe, accessible treatments for end-stage liver disease.
