# Correction: Harnessing mesenchymal stromal cells for liver disease therapy: from mechanistic discoveries to clinical breakthroughs

**DOI:** 10.3389/fmed.2026.1830211

**Published:** 2026-03-25

**Authors:** Frontiers Production Office

**Affiliations:** Frontiers Media SA, Lausanne, Switzerland

**Keywords:** clinical applications, hepatic regeneration, HSCs, immunomodulation, MSCs

Author “Jieyan Wang” was erroneously omitted as a corresponding author. The correct corresponding authors are “Yantong Wang, Xinjiang Hou, and Jieyan Wang.”

The original version of this article has been updated.

